# Dr. Bernard F. Dennis

**Published:** 1913-10

**Authors:** 


Dr. Bernard F. Dennis, Buffalo, 1899, died in Oil City July 31, aged 36. He was born in Buffalo January 8, 1877, and resided in that city till after his graduation in medicine. After a year's interne service at St. Mary's Hospital, Rochester, he located in Niagara Falls and practiced there for eight years. He then went to Europe, studying in Vienna and Berlin, meeting in the latter place the musician, Miss Bertha Snyder, who later became his wife. On his return to this country he opened an office in Buffalo, maintaining also his office in Niagara Falls. The strain of professional work gradually undermined his health to a more serious degree than anyone but himself realized. About ten days before his death he left for a rest. The funeral was held at the home of his mother in Charlotte, N. Y.

